# Neurovascular injuries associated with proximal humerus fractures: a review of the current literature

**DOI:** 10.1016/j.xrrt.2026.100825

**Published:** 2026-07-29

**Authors:** Cagman Seker, Andrea Estfeller, Ali Geçer, Agahan Hayta, Rony-Orijit Dey Hazra, David Alexander Back, Doruk Akgun, Alp Paksoy

**Affiliations:** a Center for Musculoskeletal Surgery, Charité-Universitätsmedizin Berlin, Corporate Member of Freie Universität Berlin and Humboldt-Universität zu Berlin, Berlin, Germany; b Medical University of Vienna, Vienna, Austria; c Department of Orthopedics and Traumatology, Haydarpasa Numune Training and Research Hospital, İstanbul, Türkiye

**Keywords:** Proximal humerus fracture, Trauma, Neurovascular injury, Axillary artery, Brachial plexus, Axillary nerve, Diagnostic algorithm, Treatment protocol

## Abstract

Neurovascular injuries associated with proximal humerus fractures represent a rare yet clinically significant complication with potential for devastating functional outcomes. Their multifactorial etiology encompasses direct trauma from displaced fracture fragments, indirect stretch or compression mechanisms, and iatrogenic causes during surgical management. The close anatomic relationship between the proximal humerus, axillary artery, and brachial plexus predisposes these structures to combined injury patterns that can threaten limb viability. Diagnosis relies on early recognition through meticulous clinical examination and advanced imaging modalities, which enable precise localization and characterization of neurovascular compromise. While most nerve injuries, particularly involving the axillary nerve, demonstrate favorable outcomes with conservative management, vascular injuries demand urgent multidisciplinary intervention to restore perfusion and prevent irreversible ischemia. Despite advances in imaging and endovascular techniques, no standardized management algorithm exists to universally optimize outcomes in these complex cases. This narrative review provides a comprehensive synthesis of current evidence regarding the diagnosis and management of neurovascular injuries associated with proximal humerus fractures, highlights early recognition and coordinated multidisciplinary care as key determinants of outcome and proposes a pragmatic diagnostic and treatment framework to support clinical decision-making.

Neurovascular injuries represent one of the most serious complications associated with proximal humerus fractures (PHFs), carrying significant potential for long-term functional impairment and disability.^14^ The intimate anatomic relationship between the proximal humerus and critical neurovascular structures, notably the axillary nerve, the brachial plexus, and the axillary artery with its branches, renders these elements particularly susceptible to damage at the time of injury and during subsequent surgical management.^8^^,^^31^^,^^71^^,^^72^ Although the absolute incidence of clinically relevant neurovascular compromise after PHF is low, the consequences of delayed recognition or suboptimal treatment can be catastrophic, ranging from persistent motor and sensory deficits to limb-threatening ischemia.

The epidemiology of PHFs is shifting in parallel with demographic change, as an aging population and increasing osteoporosis prevalence continue to drive fracture incidence.^37^^,^^38^^,^^51^^,^^52^ PHFs account for approximately 5% of all fractures and nearly half of all humeral fractures, with the greatest burden borne by women older than 60 years.^4^^,^^8^^,^^35^ The distribution of injury mechanisms is bimodal, low-energy falls predominate in older female patients, whereas younger male patients more often sustain high-energy trauma.^3^^,^^8^^,^^39^ Concurrently, diagnostic and care pathways for associated neurovascular injury have become more sophisticated; however, standardized diagnostic and management strategies remain lacking because of the rarity and heterogeneity of these injuries. High-resolution imaging modalities, particularly computed tomography angiography (CTA) and advanced magnetic resonance imaging (MRI),^38^ have improved detection and anatomic delineation of vascular and neural lesions, while an integrated, multidisciplinary approach involving orthopedic, vascular, and neurologic specialists has enhanced coordination of care and clinical outcomes.^46^ This review therefore synthesizes contemporary evidence regarding the presentation, diagnosis, and management of neurovascular complications following PHFs and proposes pragmatic, evidence-based diagnostic and treatment algorithms to guide clinicians.

## Neurovascular anatomy

**Figure 1:**
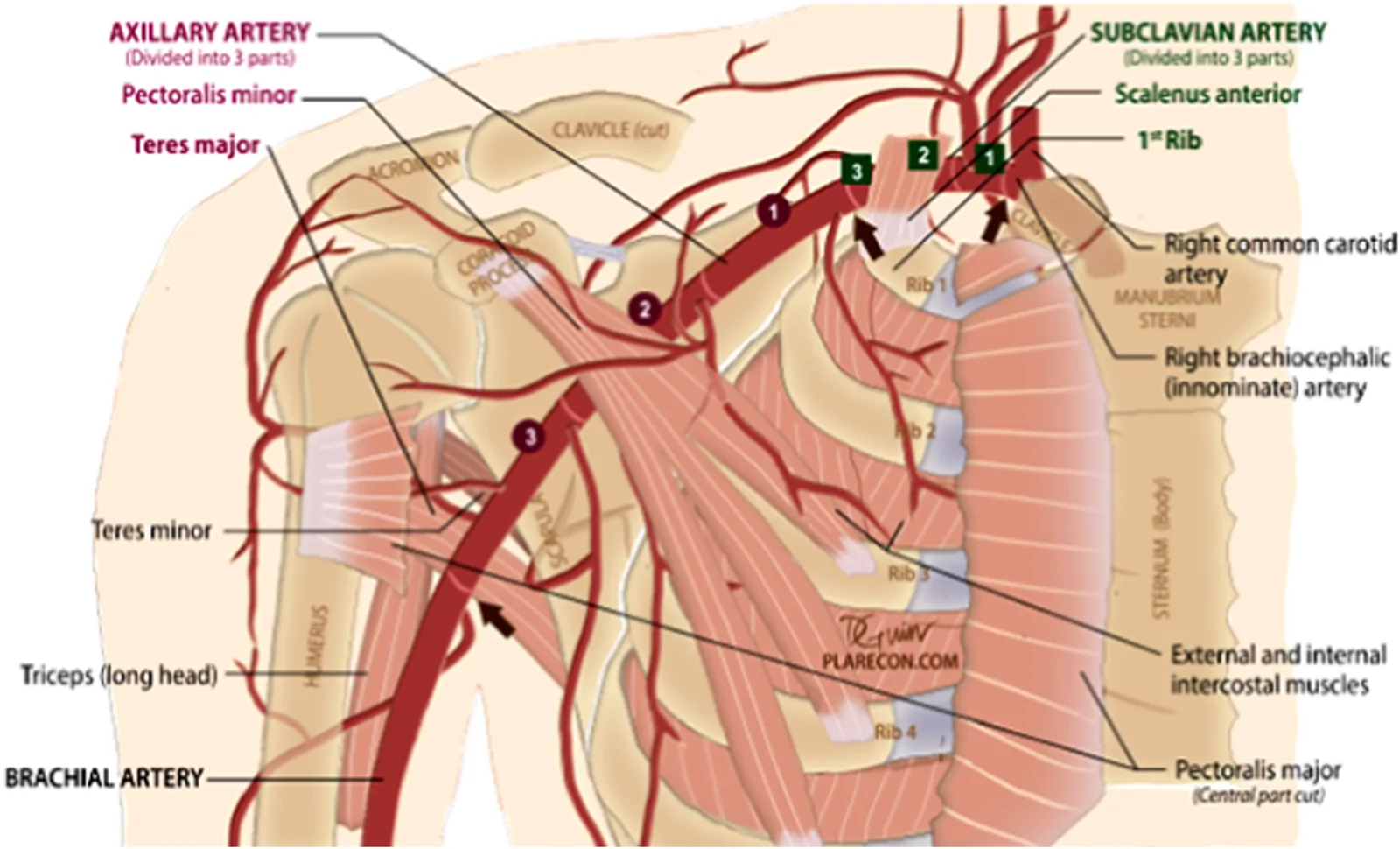
Schematic representation of the axillary artery in the right shoulder, illustrating its anatomic boundaries and the 3 segments defined in relation to the pectoralis minor muscle. Illustration by Dr. Devajyoti Guin, used with permission from www.plarecon.com.^18^

The intricate spatial relationship between the proximal humerus and adjacent neurovascular structures creates a tightly confined three-dimensional corridor in which traumatic forces may compromise multiple anatomic systems simultaneously.^52^ The axillary artery courses through the axilla in close apposition to the surgical neck of the humerus, rendering it particularly vulnerable in the setting of high-energy mechanisms and markedly displaced fractures.^79^ Classically, its trajectory is divided into 3 segments based on its relation to the pectoralis minor muscle, with injuries most frequently involving the third segment, which lies closest to common fracture lines in PHFs (Fig. 1).^60^

**Figure 2:**
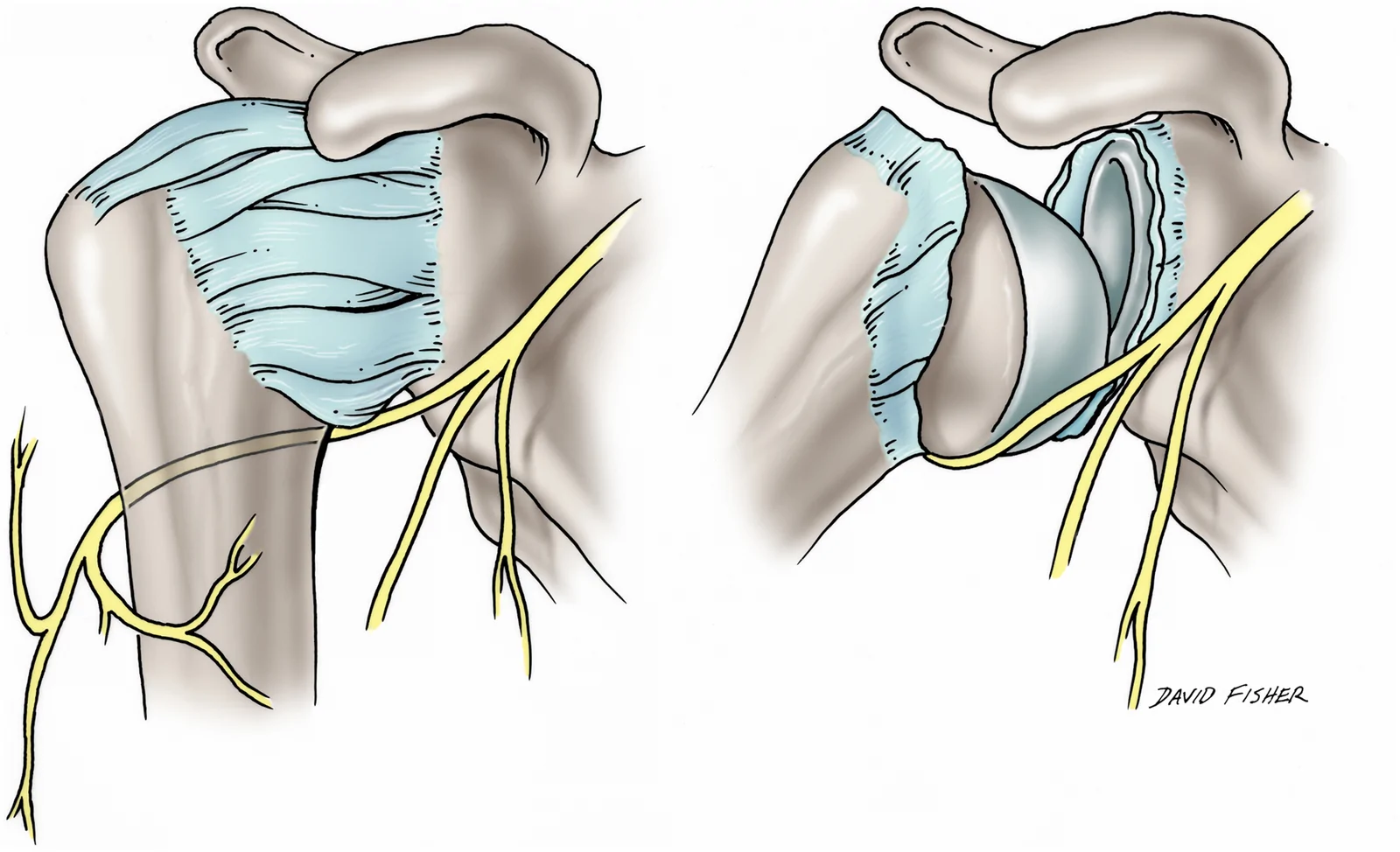
Illustration of the axillary nerve anatomy and its typical injury pattern in shoulder fracture-dislocation, demonstrating its course around the surgical neck of the humerus and its proximity to displaced fragments. Adapted with permission from Apaydin et al (2010).^1^

**Figure 3:**
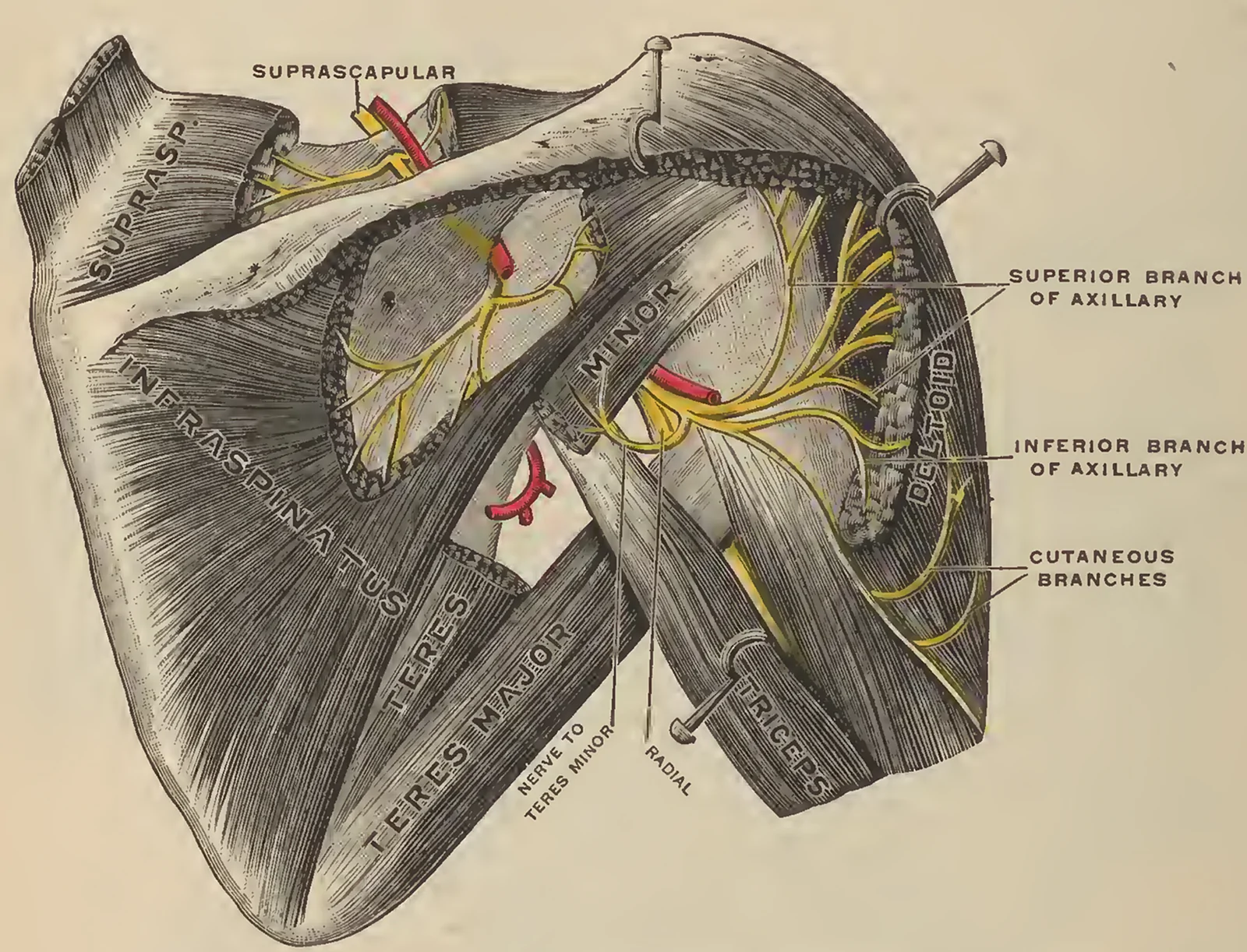
Illustration of the Right shoulder posteriorly demonstrating the regional neurovascular anatomy and the course of the axillary nerve through the quadrangular space adjacent to the surgical neck of the humerus. Reproduced from Gray's Anatomy (public domain), via Wikimedia Commons.^21^

The brachial plexus, composed of the C5–T1 nerve roots, traverses the axillary region in intimate proximity to both the axillary artery and the proximal humerus (Fig. 2).^47^ Among its terminal branches, the axillary nerve, originating from the posterior cord (C5–C6), is the neural structure most commonly affected in PHFs, a vulnerability dictated by its characteristic course around the surgical neck of the humerus (Fig. 2).^76^ As it passes through the quadrangular space, defined by the teres minor superiorly, teres major inferiorly, the long head of the triceps medially, and the humeral shaft laterally, the nerve is uniquely susceptible to traction, contusion, or laceration in the context of fracture displacement or during surgical exposure (Fig. 3).^10^ This close anatomic relationship explains the characteristic pattern of neurovascular injuries observed in PHFs, particularly in displaced fractures and fracture-dislocations.

### Mechanisms of neurovascular injury

Neurovascular compromise associated with PHFs arises through several distinct mechanisms, each characterized by specific patterns of tissue damage and clinical manifestations.^77^ Direct mechanisms involve immediate mechanical insult to vessels or nerves through compression, laceration, or avulsion by displaced bone fragments or by the primary traumatic force itself.^48^ These injuries are particularly prevalent in high-energy settings, including motor vehicle collisions, falls from height, and ballistic trauma, where substantial force transmission can produce instantaneous vascular disruption or complete nerve avulsion.^41^

Indirect mechanisms encompass secondary processes such as compression from expanding hematoma, traction injuries resulting from fragment displacement, and ischemia attributable to arterial compromise.^45^ Large fracture fragment displacement, associated hematoma formation, and advanced age have been associated with increased rates of axillary nerve injury.^72^ Furthermore, severely displaced three- and four-part fractures, fracture-dislocations, and extensive deltoid-splitting approaches may increase the risk of iatrogenic neurovascular injury because of distorted anatomy and extensive soft-tissue manipulation.^10^^,^^42^^,^^43^^,^^73^

## Classification systems for nerve injuries

Understanding nerve injury classification systems is fundamental to guiding management and predicting outcomes of neurovascular complications in PHFs. Two primary classification systems guide clinical decision-making and treatment planning.

### Seddon classification system

The Seddon classification provides a foundational framework for understanding peripheral nerve injuries based on anatomic and functional considerations. This system categorizes nerve injuries into 3 primary types (Table I).^53^

**Table I:** Seddon classification of nerve injuries.

Grade	Type	Pathophysiology	Prognosis	Recovery time
I	Neurapraxia	Temporary conduction block without structural damage	Excellent	Days to weeks
II	Axonotmesis	Axonal disruption with intact endoneurium	Good	Months
III	Neurotmesis	Complete nerve transection	Poor without surgical repair	Variable, often incomplete

Neurapraxia represents the mildest form of nerve injury, characterized by temporary conduction block without structural damage to the nerve fiber.^27^ This typically results from compression or mild stretch injury and demonstrates excellent recovery potential, usually within days to weeks. Axonotmesis involves disruption of axons while maintaining the integrity of surrounding connective tissue structures, including the endoneurium, perineurium, and epineurium.^62^ Recovery occurs through axonal regeneration at approximately 1 mm per day,^54^ with generally favorable outcomes. Neurotmesis represents complete nerve transection with disruption of all supporting structures, typically requiring surgical intervention for meaningful recovery.^55^ However, in the acute trauma setting, accurate differentiation between intermediate injury grades may be difficult because pain, swelling, and limited clinical examination can obscure the true degree of neural damage.

### Sunderland classification system

The Sunderland classification provides a more detailed and clinically relevant framework by expanding the original Seddon system into 5 distinct grades.^62^ This classification offers greater precision in injury assessment and treatment planning (Table II).

**Table II:** Sunderland classification of nerve injuries.

Grade	Anatomic damage	Clinical features	Prognosis	Treatment
I	Myelin damage only	Temporary weakness/numbness	Excellent, complete recovery	Conservative
II	Axon damage, endoneurium intact	Motor/sensory loss	Good, functional recovery	Conservative
III	Axon and endoneurium damage	Complete motor/sensory loss	Variable, may have residual deficit	Conservative initially
IV	Axon, endoneurium, and perineurium damage	Complete loss, poor recovery	Poor without intervention	Surgical repair
V	Complete transection	Complete loss	Poor without intervention	Surgical repair/reconstruction

The Sunderland system's clinical utility lies in its ability to guide treatment decisions and provide more accurate prognostic information.^43^ Grades I and II injuries typically recover with conservative management, while grades IV and V generally require surgical intervention. Grade III injuries represent a challenging intermediate category where initial conservative management with careful monitoring is appropriate, with surgical intervention reserved for cases showing no recovery after 3 to four months.^5^ In clinical practice, the Seddon classification is commonly used for the initial assessment of nerve injuries because of its simplicity, whereas the Sunderland classification provides greater detail for prognostic evaluation and surgical decision-making in more complex cases.

## Diagnostic approaches and clinical assessment

### Initial clinical evaluation

A systematic, time-sensitive clinical assessment is mandatory in any patient with a PHF in whom neurovascular injury is suspected, as delayed diagnosis or treatment may result in irreversible sequelae, including permanent neurologic deficit or limb-threatening ischemia.^58^^,^^78^ The clinical examination should be deliberately structured to detect both vascular and neural compromise and to triage patients to urgent imaging or operative intervention when indicated. The relevance of this structured approach is reflected in the published literature. Table III summarizes reported case series and case reports, illustrating the spectrum of mechanisms, presentations, imaging findings, interventions, and outcomes. Collectively, these reports demonstrate that absent pulses, expanding hematoma, progressive neurologic deficits, or signs of limb ischemia should prompt immediate vascular assessment and multidisciplinary management. The axillary artery is the most frequently implicated vessel, with injury patterns that include intimal tear and thrombosis, rupture, dissection, and pseudoaneurysm formation.

**Table III:** Summary of reported 16 cases of proximal humerus fractures associated with neurovascular injury, including clinical presentation, management, and outcomes.^9^^,^^11^, ^12^, ^13^^,^^19^^,^^20^^,^^23^^,^^25^^,^^36^^,^^41^^,^^44^^,^^45^^,^^57^^,^^63^^,^^64^^,^^67^

Author (yr)	Mechanism and fracture classification	Clinical presentation	Imaging and diagnosis	Management	Complications/Outcome
Bucci (2017)^9^	Fall on outstretched arm (Neer two-part fracture)	Marked neuro deficit, hematoma	None initially	Expanded PTFE graft bypass	Successful bypass, intact nerves
Cawich (2015)^11^	Fall on arm	Cold limb, absent pulses	Doppler US	Gore-Tex graft bypass	Successful revascularization
Chen (2018)^12^	Motorcycle accident	No blood pressure or oxygen in left upper limb	CTA, Doppler US	End-to-end anastomosis, fracture fixation	Artery shortened, good outcome
Cotman (2017)^13^	Low-energy fall (two-part fracture-dislocation)	Pale hand, absent distal pulses	Doppler US, CTA	Reverse saphenous vein graft	Successful bypass, improved perfusion
Irimia (2020)^23^	Fall from 5m (bilateral PHFs and dislocations)	Absent right arm pulses, cold/pale limb	Doppler US, perioperative arteriography	Stent + bypass graft, ORIF	Restored flow, good perfusion
Di Giacomo (2021)^19^	Domestic fall (4-part fracture-dislocation)	Warm hand but absent radial pulse	Doppler US, CTA	Saphenous vein graft + arthroplasty	Good recovery
Githens (2018)^20^	Crushed by falling pallets (bilateral PHF-dislocation)	Massive hemorrhage, diminished pulses	CTA	Hemiarthroplasty + End-to-end anastomosis	Complex case, staged procedures
Kurnaz (2018)^25^	Minor trauma (segmental dissection)	Absent distal pulses	CTA	PTFE interposition graft + resection arthroplasty	Survived with good perfusion
Palanisamy (2017)^36^	Heavy iron gate trauma (4-part fracture)	Feeble pulse, paresthesia	Doppler US, CTA	Temporary fixation, pulse restored	Temporary fixation, functional recovery
Peters (2017)^41^	Multiple falls (6 cases, mixed fracture patterns)	Multiple cases with absent pulses, large hematomas	CTA	Multiple interventions (ORIF, stenting, shunt, PTFE interposition graft.)	Mixed outcomes, 1 death
Rajeev (2019)^44^	Fall (anterior dislocation, comminuted fracture)	Pseudoaneurysm signs, posterior cord palsy	CTA	Stenting, antibiotics	Improved, died from Myocardial Infarction
Razaeian (2020)^45^	Fall (displaced multifragment PHF)	Pallor, prolonged capillary refill	CTA	Reverse arthroplasty + vein graft	Good perfusion post-op
Shieh (2022)^57^	E-bike accident (proximal humerus and olecranon fracture)	Normal initial exam, displaced humeral head	CT	Deltopectoral ORIF, posterior olecranon repair, pulse restored	Avascular Necrosis, healing
Sungelo (2019)^63^	Fall with 4-part fracture, glenoid fracture	Enlarging mass, radial nerve deficit	X-ray, CTA	Stent repair + reverse arthroplasty	Pseudoaneurysm, improved function
Thorsness (2014)^64^	Various falls (3 cases with mixed fracture-dislocations)	Diverse neurovascular deficits	X-ray, Doppler US	Hemiarthroplasty, bypass grafts	One death, 1 bypass, 1 plexopathy
Usman (2019)^67^	Motorbike fall (PHF)	Delayed ischemic signs	Doppler US, CTA	ORIF + vein graft bypass	Good recovery, radial pulse restored

*CT*, computed tomography; *CTA*, computed tomography angiography; *PTFE*, polytetrafluoroethylene; *US*, ultrasound; *PHF*, proximal humerus fracture; *ORIF*, open reduction and internal fixation.

Vascular assessment begins with careful palpation of peripheral pulses (brachial, radial, and ulnar), while acknowledging that palpable distal pulses do not exclude significant axillary arterial injury owing to robust collateral circulation.^74^ Documented signs of vascular compromise include absent or diminished pulses, prolonged capillary refill (>2 seconds), regional coolness, pallor or cyanosis, and an expanding or pulsatile hematoma. Classic “hard” signs (absent pulse with ischemia, expanding hematoma, active hemorrhage) mandate immediate surgical exploration, whereas “soft” signs (diminished pulse, bruit, or disproportionate swelling) should prompt expedited vascular imaging.^13^ The detection of a palpable thrill or audible bruit should raise suspicion for pseudoaneurysm formation.^56^

**Figure 4:**
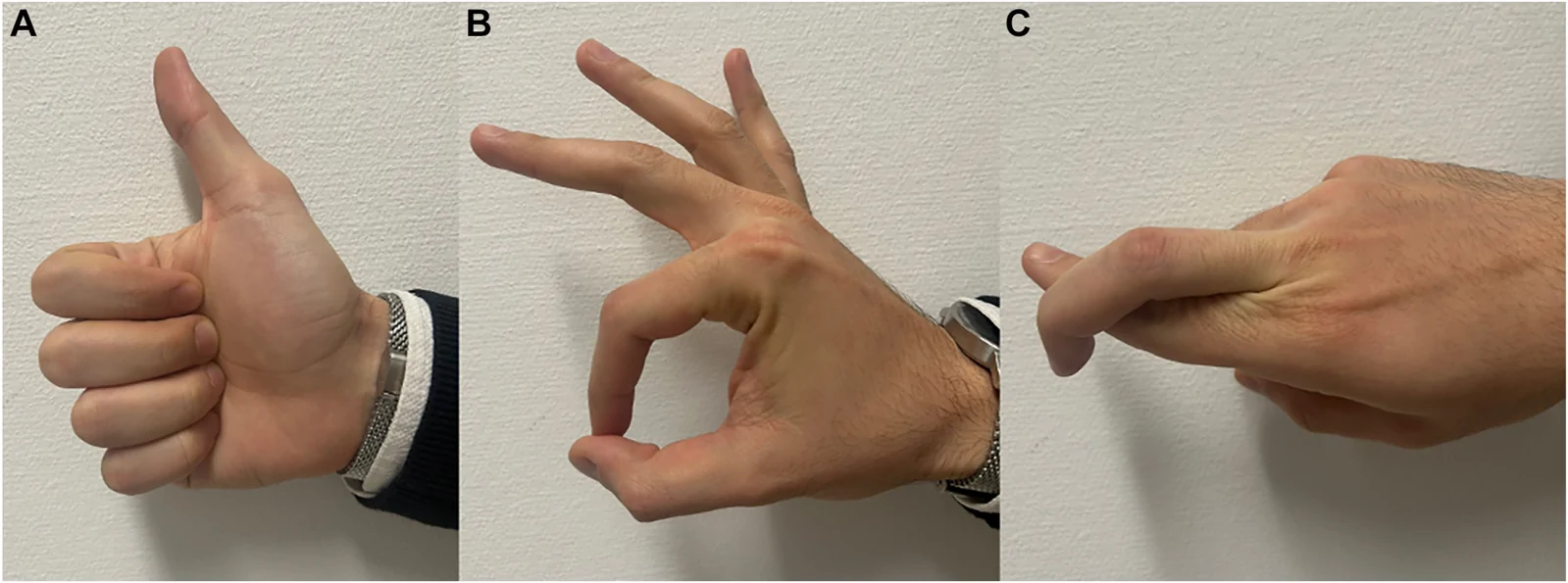
Clinical hand examination maneuvers used to assess peripheral nerve function in proximal humerus fractures. (**A**) Thumb or wrist extension testing for radial nerve integrity. (**B**) Formation of the “OK-sign” assessing median nerve motor function. (**C**) Finger-crossing maneuver evaluating ulnar nerve–mediated intrinsic muscle activity.

Neurologic assessment requires a targeted motor and sensory examination of nerves at risk, with particular attention to axillary nerve function.^40^ Axillary nerve testing includes deltoid strength assessment in its 3 distinct sets of muscle fibers (pars clavicularis/scapularis/acromialis) and sensory evaluation over the lateral shoulder (“regimental badge” area).^6^ Because reliable isolated rotator-cuff testing (supraspinatus/infraspinatus) may be difficult immediately after injury, distal neurologic assessment is often more practicable and informative.^66^ Motor and sensory testing of the radial, median and ulnar nerves should be prioritized to screen for brachial plexus involvement and to identify deficits suggestive of major nerve injury or limb ischemia. Thumb or wrist extension assesses radial nerve function, forming an “OK-sign” evaluates median nerve integrity, and finger crossing tests ulnar nerve–mediated intrinsic muscle function (Fig. 4). These simple maneuvers help detect early motor deficits and establish a dependable baseline for follow-up examinations. Even when an initial examination is limited by pain or swelling, serial examinations are essential to define the trajectory of recovery or deterioration.^17^^,^^33^^,^^49^^,^^66^^,^^73^

### Imaging modalities and diagnostic tests

Modern imaging is integral to the evaluation of suspected neurovascular injury after PHF and should be selected according to clinical suspicion and the diagnostic question at hand.^2^ Initial radiographic assessment should include orthogonal plain films (true anteroposterior and Y- or axillary lateral views) to define fracture pattern and identify frank dislocation. When fracture-dislocation or clinical features suggest vascular compromise, CTA is indicated without delay. However, its use may be limited in elderly patients with renal impairment or in settings with limited immediate availability. Duplex ultrasonography can serve as a rapid bedside screening modality but is often limited acutely by hematoma formation and operator dependence. Computed tomography (non-contrast) is useful for detailed assessment of complex fracture morphology to inform surgical planning, while MRI is reserved for selective indications such as suspected rotator cuff tears or brachial plexus lesions rather than routine acute assessment.^78^

#### Vascular imaging

**Figure 5:**
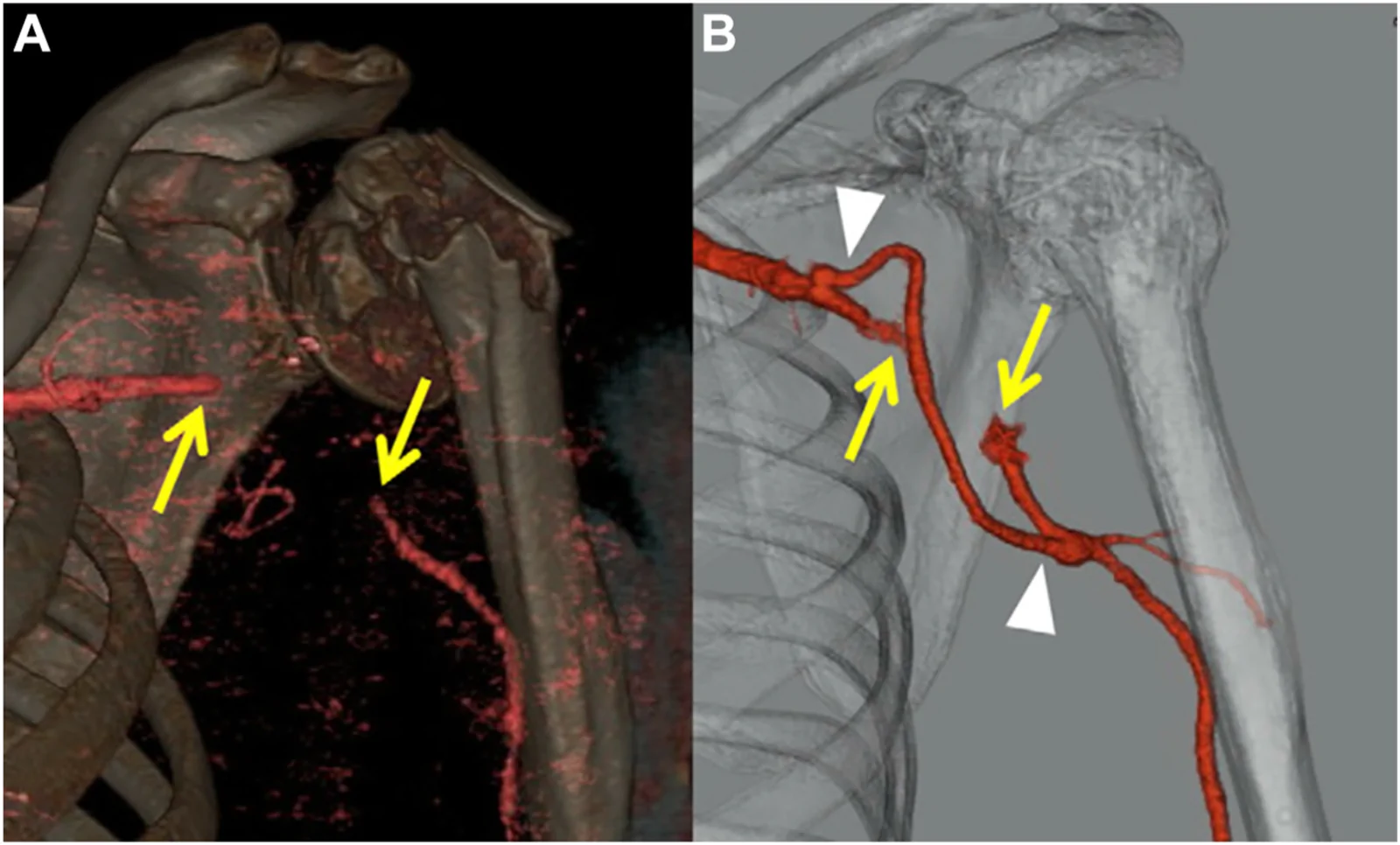
(**A**) Adapted from Mitsuzawa et al, 2024. Three-dimensional CTA demonstrating axillary artery disruption (*yellowarrows*) in a 69-year-old male who sustained a low-energy fall while intoxicated. The patient presented with severe shoulder pain, a cold hand with weak radial pulse and motor–sensory deficits in the radial and median nerve distributions. Radiographs and CT imaging revealed a three-part PHF. CTA showed a contrast defect of the axillary artery at the third segment, while distal vessel opacification was preserved through collateral circulation. (**B**) Post-operative CTA demonstrating restoration of axillary artery continuity following surgical repair. Five days after the injury, the patient underwent hemiarthroplasty (HA), after which the injured segment of the axillary artery was bypassed using a vein graft with an end-to-side anastomosis (*white arrowheads*), and neurolysis of the brachial plexus was performed.^29^*PHF*, proximal humerus fracture; *CTA*, computed tomography angiography.

Duplex ultrasound provides real-time flow assessment and can rapidly identify flow compromise,^61^ but CTA has become the diagnostic standard for detailed vascular mapping in the trauma setting because it simultaneously delineates arterial anatomy and fracture configuration (Fig. 5).^68^ Conventional angiography retains an important role when endovascular therapy is contemplated, as it allows for both definitive diagnosis and immediate therapeutic intervention.^24^

#### Neurologic assessment

High-resolution MRI offers superior soft tissue contrast and may visualize nerve edema, discontinuity, or surrounding hematoma when brachial plexus injury is suspected, but it is not routinely performed in the immediate post-traumatic phase. Electrodiagnostic studies, including electromyography (EMG) and nerve conduction studies (NCSs), are invaluable for quantifying nerve injury severity and monitoring recovery; however, because their diagnostic yield is limited during the first weeks after injury, they are typically performed 2–4 weeks post-trauma to allow for Wallerian degeneration and reliable assessment of axonal loss and reinnervation.^5^^,^^70^

## Treatment algorithm and management of neurovascular injuries

Initial management is dictated by the presence of life- or limb-threatening pathology and follows a staged, multidisciplinary approach. The overarching objective is prompt identification of injuries that require urgent vascular or neural intervention while instituting appropriate monitoring and conservative measures for less severe presentations.^16^

**Figure 6:**
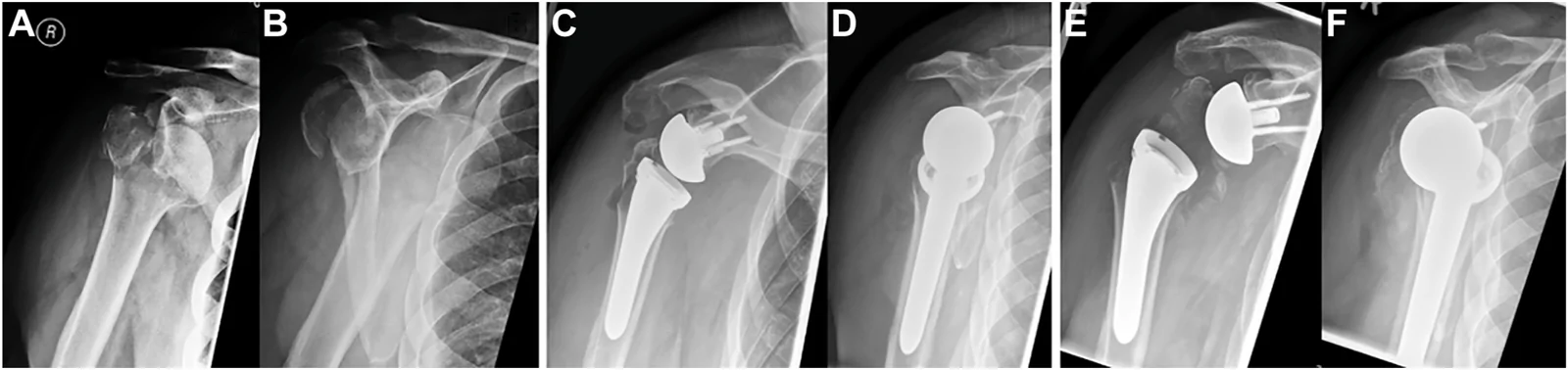
Radiographs of a 75-year-old male patient who sustained a PHF. The first images show the pre-operative fracture configuration. The patient was treated with rTSA. Subsequent images demonstrate post-operative positioning and later prosthetic dislocation, which occurred due to axillary nerve injury and resulting deltoid insufficiency. *PHF*, proximal humerus fracture; *rTSA*, reverse total shoulder arthroplasty.

Surgical fixation is indicated for displaced, comminuted, or significantly angulated fractures in appropriately selected patients.^50^^,^^69^ Early anatomic reduction of the humeral head and stable fixation optimize the mechanical environment for recovery. Operative options include open reduction and internal fixation, intramedullary nailing, hemiarthroplasty (HA), reverse total shoulder arthroplasty, or closed reduction with percutaneous pinning depending on fracture morphology, patient factors and the presence of associated neurovascular compromise.^22^^,^^74^ In the setting of fracture-dislocation with concomitant axillary nerve palsy, primary arthroplasty may be ill-advised because deltoid dysfunction substantially increases the risk of post-operative instability (Fig. 6). Therefore, careful assessment of axillary nerve function is essential before considering primary reverse total shoulder arthroplasty in complex fracture patterns. In such cases, open reduction and internal fixation with anatomic tuberosity reconstruction is often favored as the initial strategy, preserving the option for delayed arthroplasty should reconstruction fail or rotator cuff function remain inadequate.^30^^,^^34^

### Management of vascular injuries

Management requires early collaboration with vascular surgery and interventional radiology. The decision algorithm is guided by the severity of vascular compromise (hard versus soft signs), anatomic location, and patient comorbidity.^15^ Hard signs mandate emergent exploration and repair, possibly including temporary shunting when prolonged ischemia is anticipated. Soft signs generally prompt CTA or diagnostic angiography for clarification. In the acute phase, intimal injuries pose a high thrombotic risk, whereas pseudoaneurysms often present subacutely (weeks to months after the initial trauma).^29^

Contemporary management increasingly incorporates endovascular techniques for selected vascular injuries.^59^ Covered stent grafts have shown excellent results for focal arterial injuries, while embolization techniques are effective for bleeding from smaller branch vessels.^75^ Furthermore, direct repair or interposition grafts are preferred over bypass grafts for the management of vascular injuries associated with fractures according to current guidelines, as these approaches may reduce both morbidity and potential mortality.^7^ Temporary arterial shunting should also be considered when prolonged ischemia is anticipated or fracture fixation may delay definitive vascular repair, as early restoration of perfusion remains critical for limb salvage.^7^^,^^57^

## Management of nerve injuries

Management is determined by the injury grade and the anticipated potential for spontaneous recovery. Most traumatic nerve injuries in this context are managed initially with observation and supportive care, reserving surgical intervention for specific indications.^65^ When managing nerve injuries conservatively, several key steps should be carefully followed throughout the recovery process. In the acute phase, joint protection and gentle range-of-motion exercises should be initiated to maintain mobility and prevent stiffness.^62^

Sensory nerve action potentials typically begin to decrease by day 5 and reach their lowest by day 11, while motor amplitudes fall by day 3 and reach their lowest by day 7,^28^ significantly limiting the diagnostic value of electrodiagnostic studies in the immediate postinjury period. For this reason, EMG/NCS is recommended to be performed 2 to four weeks after injury, particularly when neurologic deficits fail to show clinical improvement, as this timing allows for a more reliable evaluation of axonal damage.^65^^,^^74^ Between 3 and six months, serial clinical and electrodiagnostic follow-up should be performed to monitor nerve regeneration. By 6 to twelve months, surgical intervention should be considered if no functional recovery is evident. During this period, electrodiagnostic testing, especially EMG, helps quantify reinnervation and guides the decision-making process regarding the need for surgical exploration.

Surgical management is reserved for cases in which spontaneous recovery is unlikely or further deterioration is expected. These include complete nerve transection (Sunderland Grade V) injuries, lack of clinical improvement after 3 to four months in cases of Grade IV lesions, progressive neurologic decline during follow-up and open wounds with visible nerve disruption. Timely operative repair is essential to optimize functional outcomes, as prolonged delays may result in irreversible denervation atrophy and markedly reduced reinnervation potential.^26^

Primary nerve procedures include neurolysis, direct repair, nerve grafting, or nerve transfers. In complex cases with loss of muscle function, adjunctive or staged procedures, such as tendon transfers, free functional muscle transfer, or osseous realignment, may be necessary to restore function. Nerve grafting or transfers are generally less favorable beyond 12 months postinjury because reinnervation potential declines with time.^32^

**Figure 7:**
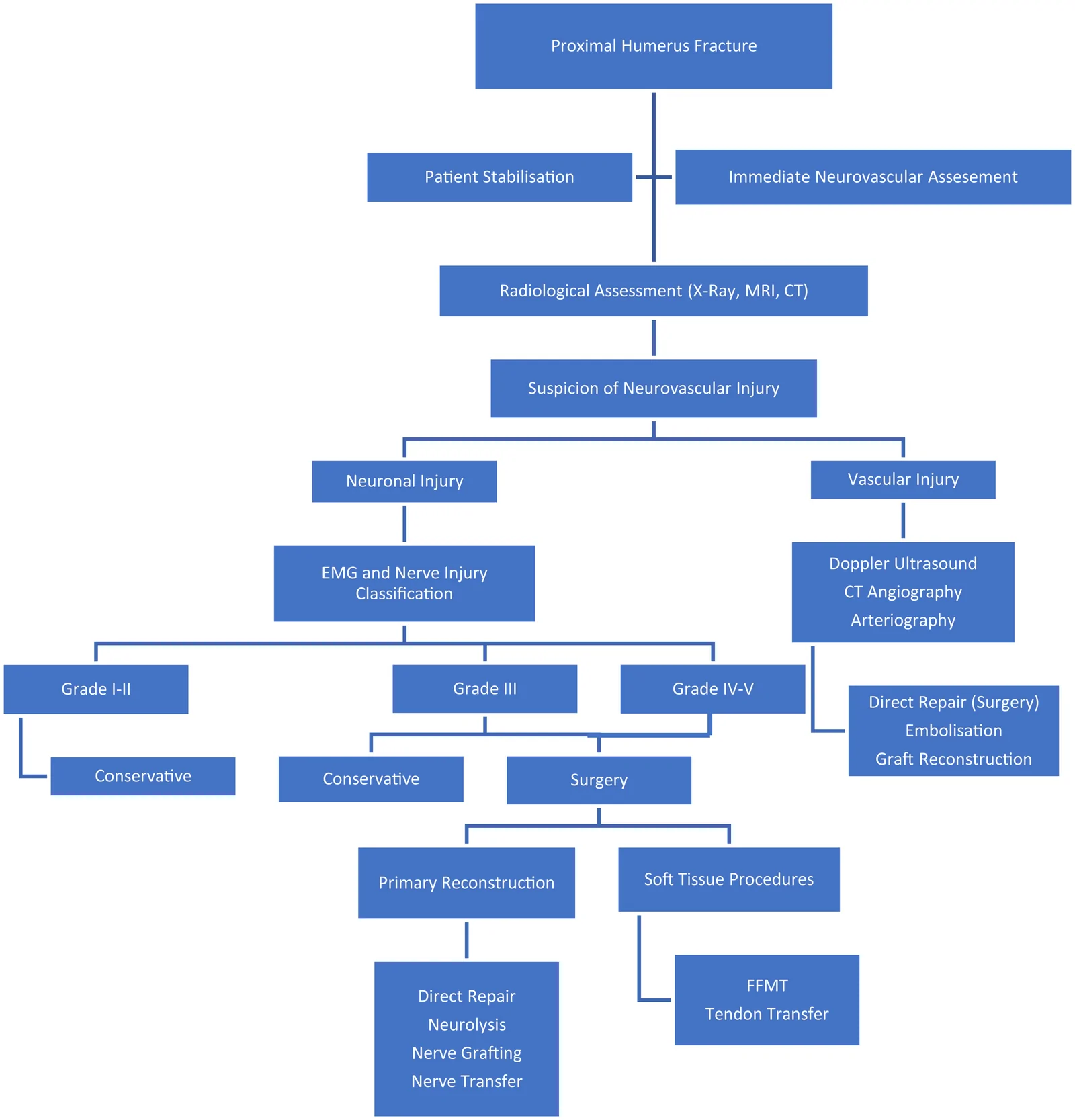
Diagnostic algorithm for neurovascular injuries. *EMG*, electromyography; *CT*, computed tomography, *MRI*, magnetic resonance imaging; *FFMT,* free functional muscle transfer.^15^^,^^16^^,^^26^^,^^29^^,^^32^^,^^65^^,^^74^

A diagnostic algorithm for the assessment and management of neurovascular injuries associated with PHFs was developed by the authors based on the available literature and is summarized in Figure 7 as a pragmatic framework to support multidisciplinary clinical decision-making.

## Discussion

Neurovascular injuries associated with PHFs remain rare but potentially devastating complications, with increasing clinical relevance due to the rising incidence of PHFs. Their consequences may range from neurologic deficits and limb ischemia to severe functional impairment. Therefore, maintaining a high index of suspicion during the acute phase remains essential for prompt diagnosis and management. Diagnosis may be challenging because swelling, hematoma, pain, and concomitant injuries may mask clinical findings at initial presentation. However, delayed diagnosis and treatment may negatively affect functional outcomes, patient satisfaction and quality of life. Therefore, targeted clinical and radiological assessment remains essential during the initial evaluation.

The complications associated with PHFs may present through a wide spectrum of injury mechanisms and clinical manifestations, ranging from direct vascular or nerve disruption to more subtle secondary injuries caused by hematoma formation, traction, or ischemia. Furthermore, potentially misleading findings such as preserved distal pulses due to collateral circulation, swelling, hematoma formation, pain and delayed neurologic deficits may further obscure the diagnosis and contribute to delayed recognition. Even with the availability of high-resolution imaging modalities, neurovascular injuries may remain undiagnosed if targeted diagnostic investigations are not performed. The rarity and heterogeneity of these injuries, combined with the lack of standardized diagnostic and management strategies, continue to represent a challenge in both the clinical practice and literature. Therefore, despite their low incidence, a structured and standardized approach remains essential in the presence of high-energy trauma, three- or four-part displaced fractures, fracture-dislocations, or suspicious clinical findings. Such an approach may facilitate timely diagnosis, help exclude clinically relevant injuries and support decision-making in daily practice. The cases summarized in Table III further illustrate the heterogeneous presentation and management of neurovascular injuries, particularly with regard to associated neurologic involvement and diagnostic work-up.

Despite the variability in clinical presentation, absent pulses, progressive neurologic deficits, expanding hematoma, and signs of limb ischemia consistently emerged as important warning signs requiring urgent vascular assessment and multidisciplinary management. In most reported cases, CTA was performed and proved valuable for confirming the diagnosis and guiding further treatment. Given the close anatomic relationship between the axillary artery and the proximal humerus, the axillary artery was the most frequently affected vessel. The broad spectrum of vascular injury patterns, including thrombosis, intimal tears, dissection, rupture, and pseudoaneurysm formation, may also explain the variability of the surgical and endovascular treatment strategies reported in the literature.

Although clinical manifestations varied considerably and vascular reconstruction was frequently required, the reviewed cases also demonstrated that vascular compromise often coexisted with neurologic injury, with concomitant neurologic involvement reported in 14 of 23 vascular injuries (61%). This finding further highlights the close anatomic and clinical relationship between vascular and neural structures around the proximal humerus and underscores the importance of a multidisciplinary approach, including thorough neurologic assessment and, when indicated, early neurologic consultation.

Considering the close anatomic relationship between the axillary nerve and the surgical neck of the humerus, axillary nerve dysfunction represented the most frequently reported neurologic complication. However, neurologic injuries demonstrated a broad spectrum, ranging from isolated axillary nerve deficits to more extensive brachial plexus involvement. Consequently, clinical manifestations may vary from mild paresthesia to severe motor and sensory deficits. Another challenge regarding neurologic complications is that deficits may be masked during the acute phase because of pain, swelling, and associated traumatic injuries. In addition, many neurologic deficits may partially or completely recover over time. Therefore, careful follow-up of neurologic symptoms remains essential. EMG and NCS should be performed at an appropriate interval, typically 2–4 weeks after injury, and may be repeated during follow-up up to 6–12 months after trauma. This approach may help avoid overtreatment while identifying patients who may benefit from surgical intervention.

Based on the available literature, we propose a pragmatic diagnostic and treatment algorithm in Figure 7 to facilitate the assessment and management of neurovascular injuries associated with PHFs. Nevertheless, the current evidence remains limited and is largely based on case reports and small case series. Future multicenter studies are required to better define diagnostic pathways, optimal timing of intervention and treatment strategies for these uncommon but potentially devastating injuries.

## Conclusion

A structured clinical pathway that integrates meticulous initial examination, judicious use of imaging modalities (particularly CTA), timely electrodiagnostic assessment, and collaborative decision-making between orthopedic, vascular, and neurologic teams offers the best opportunity to recognize and manage neurovascular complications of PHFs. Early, individualized intervention guided by clinical severity and diagnostic findings remains the cornerstone for preventing irreversible ischemic injury and maximizing neurologic recovery.

## Disclaimers:

Funding: No funding was disclosed by the authors.

Conflicts of interest: The authors, their immediate family, and any research foundation with which they are affiliated have not received any financial payments or other benefits from any commercial entity related to the subject of this article.
